# Energy Metabolism Disorder as a Contributing Factor of Rheumatoid Arthritis: A Comparative Proteomic and Metabolomic Study

**DOI:** 10.1371/journal.pone.0132695

**Published:** 2015-07-06

**Authors:** Xin Yu Yang, Kai Di Zheng, Ke Lin, Guifeng Zheng, Hai Zou, Jian Min Wang, Yao Yao Lin, Chifundo Martha Chuka, Ren Shan Ge, Weitao Zhai, Jian Guang Wang

**Affiliations:** 1 Department of Medicinal Chemistry, School of Pharmaceutical Sciences, Wenzhou Medical University, Wenzhou, China; 2 Department of Biochemistry, School of Basic Medical Sciences, Wenzhou Medical University, Wenzhou, China; 3 Department of Rheumatology, Jiamusi Central Hospital, Jiamusi, China; 4 Department of Orthopaedic Surgery, Shanghai Guanghua Special Hospital for Rheumatoid Arthritis, Shanghai, China; University of Nebraska Medical Center, UNITED STATES

## Abstract

**Objectives:**

To explore the pathogenesis of rheumatoid arthritis (RA), the different metabolites were screened in synovial fluid by metabolomics.

**Methods:**

Synovial fluid from 25 RA patients and 10 normal subjects were analyzed by GC/TOF MS analysis so as to give a broad overview of synovial fluid metabolites. The metabolic profiles of RA patients and normal subjects were compared using multivariate statistical analysis. Different proteins were verified by qPCR and western blot. Different metabolites were verified by colorimetric assay kit in 25 inactive RA patients, 25 active RA patients and 20 normal subjects. The influence of hypoxia-inducible factor (HIF)-1α pathway on catabolism was detected by HIF-1α knockdown.

**Results:**

A subset of 58 metabolites was identified, in which the concentrations of 7 metabolites related to energy metabolism were significantly different as shown by importance in the projection (VIP) (VIP≥1) and Student’s *t*-test (*p*<0.05). In the 7 metabolites, the concentration of glucose was decreased, and the concentration of lactic acid was increased in the synovial fluid of RA patients than normal subjects verified by colorimetric assay Kit. Receiver operator characteristic (ROC) analysis shows that the concentration of glucose and lactic acid in synovial fluid could be used as dependable biomarkers for the diagnosis of active RA, provided an AUC of 0.906 and 0.922. Sensitivity and specificity, which were determined by cut-off points, reached 84% and 96% in sensitivity and 95% and 85% in specificity, respectively. The verification of different proteins identified in our previous proteomic study shows that the enzymes of anaerobic catabolism were up-regulated (PFKP and LDHA), and the enzymes of aerobic oxidation and fatty acid oxidation were down-regulated (CS, DLST, PGD, ACSL4, ACADVL and HADHA) in RA patients. The expression of HIF-1α and the enzymes of aerobic oxidation and fatty acid oxidation were decreased and the enzymes of anaerobic catabolism were increased in FLS cells after HIF-1α knockdown.

**Conclusion:**

It was found that enhanced anaerobic catabolism and reduced aerobic oxidation regulated by HIF pathway are newly recognized factors contributing to the progression of RA, and low glucose and high lactic acid concentration in synovial fluid may be the potential biomarker of RA.

## Introduction

Rheumatoid arthritis (RA) is a chronic systemic autoimmune disease characterized by synovial inflammation and hyperplasia that induce autoantibody production and cartilage and bone destruction [1]. Activated fibroblast-like synoviocytes (FLS) in the lining layer of the synovial membrane are the dominant cell type involved in “pannus” formation and play a key role in joint destruction [2–4]. There are studies reporting that activation of the immune system requires a lot of energy in chronic inflammatory diseases including RA. Energy metabolism not only provides energy for inflammatory diseases but also controls immune responses via metabolic signals [5]. Therefore, energy metabolism may play an important role in the pathogenesis of RA and other inflammatory diseases [6]. But there is little research on body energy metabolism in RA patients. And the change law of energy metabolism related enzymes and the regulation pathway remains unclear.

Metabolites are intermediates or products of metabolism, playing numerous roles in the healthy and diseased body, ranging from regulating physiological processes to providing the building blocks for the body [7–10]. Metabolites changes in RA have been widely described mainly based on serum and urine as research subjects [11,12]. Kapoor et al [13] reported the differences in metabolic profiles in the urine of RA and psoriatic arthritis (PsA) patients during the treatment with infliximab and etanercept. Gu et al [14] described metabolic dysfunctions in RA by plasma metabolomics study. Young et al [15] demonstrated that underlying inflammatory processes drove significant changes in metabolism that could be measured in the peripheral blood. Low histidine levels have been reported in sera of RA patients [6,16], suggesting that changes in metabolites might be observable early in the development of RA.

Our previous comparative proteomics study screened out 100 differential proteins from primary cultured fibroblast-like synovial (FLS) cells of RA patients and normal subjects. GO and pathway analysis showed that the expression of many energy metabolism related enzymes underwent regular changes [17]. If changes in the expression of the metabolism-related enzymes may cause changes in the amount of the generation of metabolites, how would these changes affect the pathological process of RA? Metabolomics research could give us some clues. Synovial fluid is a viscous, non-Newtonian fluid found in the cavities of synovial joints. The main function of the synovial fluid is to minimize friction between the bones, and nourish the cartilage by supplying oxygen and nutrients to surrounding synovial tissue and cartilage, and removing carbon dioxide and metabolites from them. So changes in the metabolites of synovial membrane can be fully reflected in the synovial fluid. Evaluation of synovial fluid will not only provide a specific biomarker but also enable us to understand the progression of RA. It is difficult to obtain sufficient quantities of synovial fluid from knee joints [18], therefore the study on metabolites in synovial fluid has been greatly limited. The aim of the present study was to investigate RA-related biochemical abnormalities by analyzing the metabolic profiling of knee synovial fluid from RA patients and normal controls by GC/TOF MS, and to evaluate the diagnosing potential of metabolites and reveal the energy metabolism of RA.

## Materials and Methods

### Patient information

Knee synovial fluid was obtained from 75 RA patients (25 for identification by GC/TOF MS, 50 for verification by colorimetric assay kit) and synovial tissues were from RA patients of synovectomy or joint replacement surgery, fulfilling the 1987 American College of Rheumatology criteria for diagnosis of RA [19], and 20 normal subjects (10 for identification by GC/TOF MS, 20 for verification by colorimetric assay kit) of high-level amputations in Changhai Hospital and Guanghua Special Hospital for Rheumatoid Arthritis in Shanghai. RA patients for verification experiment were further categorized as an active RA group (n = 25) and an inactive RA group (n = 25) depending on the elevation of disease activity score in 28 joints (DAS28) (inactive RA: DAS28<3.2; active RA: DAS28>3.2) for DAS28 score correlates closely with clinical parameters of RA disease activity [20]. The clinical details of the patients are shown in S1A and S1B Table. Samples were stored at -80°C until analysis. This study was approved by Shanghai Changhai Hospital Ethics Committee (CHEC2013-194), with written informed consent obtained from all the participants concerned.

### GC/TOF MS analysis

100μl individual knee synovial fluid and 50 μl L-2-chlorophenylalanine (internal standard) were mixed in 1.5 ml tube, extracted by 0.35ml methanol, centrifuged at 10000rpm for 10 min at 4°C. Supernatant was completely dried in a vacuum concentrator. The sample was incubated by 80 μl methoxamine hydrochloride diluted with pyridine to 20 mg/ml at 37°C for 2.5 hr, added 100 μl BSTFA containing 1% TMCS(v/v), and hatched in 70°C for 1 hr, then the sample was analyzed by GC/TOF MS.

Derivative metabolite samples were analyzed by an Agilent 7890 gas chromatograph system (Agilent, USA) coupled to a Pegasus 4D TOF MS (LECO, USA). 1μl aliquot of the samples was injected into a DB-5MS capillary column coated with 5% diphenyl cross-linked with 95% dimethylpolysiloxane (30 m × 250 μm diameter, 0.25 μm thickness) (J&W Scientific, USA) for GC separation. Helium was used as the carrier gas and the flow rate through the column was 1 ml/min. The initial temperature was kept at 90°C for 2 min, then raised to 180°C at a rate of 5°C/min, and finally to 285°C at a rate of 15°C/min. The temperature for injection, the transfer line and ion source was 280°C, 270°C, and 220°C respectively. The energy was -70 eV in electron impact mode. The mass spectrometry data was acquired in full-scan mode with the m/z range of 20–600 at a rate of 100 spectra per second after a solvent delay of 492 s.

### Multivariate data analysis

Software Chroma TOF 4.3X was used to align the metabolite spectra by spectral match (≥600) and retention time match (<6s). Missing data was imputed by half of the minimal spectrum area. Then, metabolite spectra were standardized with the spectrum area of internal standard. Heat map was generated by software PermutMatrixEN. The data of individual sample (including normal and RA) were divided by the mean of normal samples. Then, the logarithm of metabolites data of samples to base 10 was used for heat map generation. The standardized data was also input to the SIMCA-P 11.0 Software package (Umetrics, Umea, Sweden) for the principal component analysis (PCA) and orthogonal partial least squares discriminant analysis (OPLS-DA). The model of OPLS-DA was validated by a default 7-round cross-validation procedure with exclusion of 1/7th samples in each round. At the same time, metabolites of VIP ≥1.00 and P<0.05 were identified as different metabolites between RA patients and normal subjects. When the mass spectral similarity between its peak and mass spectral database was larger than 700 (similarity over 1000 means exact match), metabolites were named by TurboMass 4.1.1 software (PerkinElmer Inc., USA) coupled with NIST mass spectra database and LECO mass spectra database [21]. If several peaks had the same name, only the peak with the highest similarity was used.

### qRT-PCR

Total RNA was extracted from the synovial tissues of 50 RA patients and 20 normal subjects by TRIzol regent as described by the manufacturer. Specific amplification was performed using the primers of *hif-1α*, *pfkp*, *ldha*, *cs*, *dlst*, *pgd*, *acsl4*, *hadha*, and *acadvl* genes (PCR primers used are listed in S2 Table). The relative expression of each target gene compared to *β*-actin was calculated using the 2^-ΔΔCt^. All reactions were conducted in triplicate.

### Western blot analysis

Synovial tissue homogenates of 20 RA patients and 20 normal subjects or FLS cells were mixed with lysates of RIPA buffer. Total protein (50μg per lane) was loaded onto an SDS-PAGE gel and transferred onto PVDF membranes (Millipore). The membranes were blocked and washed. Primary antibodies used included HIF-1α (Santa Cruz, sc-71247), PFKP (Abcam, ab72680), LDHA (Santa Cruz, sc-137243), CS (Santa Cruz, sc-242444), DLST (Santa Cruz, sc-242583), PGD (Abcam, ab119796), ACSL4 (Santa Cruz, sc-365230), HADHA (Santa Cruz, sc-271495), ACADVL (Santa Cruz, sc-271225) and *β*-actin (Abcam, ab6276), and then incubated with HRP-conjugated secondary antibody at 1:5000 in 5% skim milk for 1hr, washed and processed with ECL Plus western blot detection kit. The filter was then incubated with the substrate and exposed to X-ray films.

### FLS cells culture and HIF-1α knock-down

Synovial tissue was isolated enzymatically according to the method previously described [17]. All FLS cells of passages 3 to 5 were used for the experiment. HIF-1α knock-down experiments were performed by transfecting HIF-1α specific siRNA (sc-45919, Santa-Cruz, USA) or nonspecific (NS) siRNA into FLS cells using Lipofectamine 2000 (Invitrogen, USA) following manual instructions. RNA and protein in FLS cells were extracted for qPCR and western blot.of HIF-1α, PFKP, LDHA, CS, DLST, PGD, ACSL4, HADHA and ACADVL

### Quantitation of differential metabolites in synovial fluid

The concentrations of glucose and lactic acid (Cat # K606-100 and Cat# K607-100, BioVision, Milpitas, CA, USA) were measured in synovial fluid (25 inactive RA patients, 25 active RA patients and 20 normal subjects) by colorimetric assay kit as described by the manufacturer. A standard curve was prepared using known amounts of standard included in the kit. Absorbance was analyzed with the SPECTRAmax 190 (Molecular Devices, Sunnyvale) plate reader at 570nm. GraphPad Prism 6 was used for ROC analysis.

### Statistical analysis

Statistical analysis was performed using SPSS software version 17.0 (SPSS, Chicago, IL). The Shapiro-Wilk method and histograms were used to test whether the data was normally distributed. The Levene method was used to test homogeneity of variance. Two sets of data that met the normal distribution and homogeneity of variance were analyzed by unpaired Student’s t-test and Fisher's exact test to determine which mean value was significantly different (*P*<0.05). The Kruskal-Wallis and Mann-Whitney method of non-parametric test was used to compare inter-assay differences of the data that did not meet the normal distribution or the homogeneity of variance.

## Results

### Comparisons of the metabolite spectra from RA patients and normal subjects

**Fig 1:**
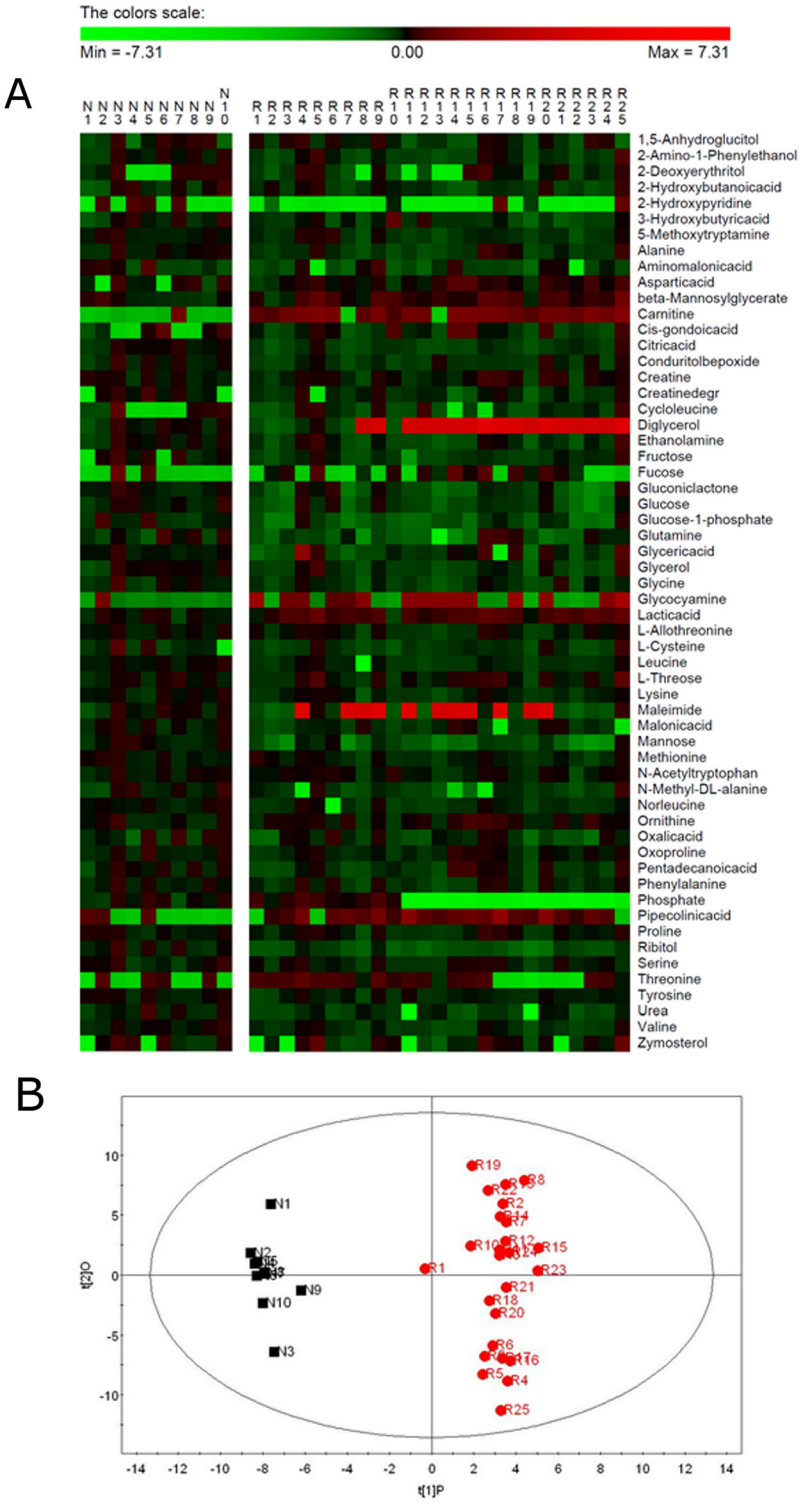
Metabolic patterns in RA patients and normal subjects. (A) The heat map shows the standard score for each metabolite of each RA patient and each normal subject. The standard score shows how the concentration of each metabolite is related to the mean value of the control group. Red color indicates that the metabolite is increased compared to the mean of the control group; green color indicates that the metabolite is decreased. Metabolites are sorted according to the initial letter of the metabolite. The standard score is truncated to -7.31/7.31 for clarity. (B) Orthogonal PLS-DA (OPLS-DA) score plot of the first two principal components of an analysis of metabolites from RA patients (R) and Normal subjects (N). The number after letter means the number of the sample. The horizontal axis and the vertical axis mean values of the first and the second principal components. The ellipse denotes the 95% significance limit of the model, as defined by Hotelling's t-test.

187 peaks were acquired using GC-MS, of them 58 metabolites were identified (Fig 1). A list of mean values and standard deviations for all identified metabolites is provided in S3 Table. Based on the full metabolic data set, two principal components were identified by PCA analysis, all samples of RA patients were evidently separated from normal group by OPLS-DA analysis (Fig 1B) with high R^2^Y value (0.965) and Q^2^Y value (0.895). The concentrations of 13 metabolites were significantly different between RA patients and normal subjects by VIP ≥ 1 and p < 0.05 (Table 1). Of the13 metabolites, 7 metabolites were related to energy metabolism, including lactic acid, valine, citric acid, gluconic lactone, glucose, glucose-1-phosphate and mannose. Of them, valine was a branched-chain acid, citric acid was from TCA cycle, and other 5 metabolites were from carbohydrate metabolism. The concentration of lactic acid was increased and that of other 6 metabolites were decreased in synovial fluid of RA patients.

**Table 1:** Different Metabolites in RA patients and normal subjects (VIP≥1, *p*<0.05).

Metabolite	Pathway^a^	R.T.^b^ (minutes)	Normal^c^	RA^c^	VIP	P value
5-Methoxytryptamine	Tryptophan metabolism	19.264	1.007±0.086	0.727±0.065	1.115	0.022
beta-Mannosylglycerate		29.545	0.556±0.098	1.272±0.201	1.269	0.035
Carnitine	Lysine degradation	16.956	0.011±0.011	0.091±0.010	2.02	<0.001
Citric acid	Citrate cycle	18.471	21.117±1.998	12.218±1.385	1.499	0.001
Diglycerol		16.77	<0.001	0.189±0.053	1.512	0.032
Gluconic lactone	Pentose phosphate pathway	20.792	7.277±0.843	4.065±0.688	1.189	0.012
D-glucose	Glycolysis/ Pentose phosphate pathway	19.545	21.640±2.950	11.846±1.741	1.187	0.006
Glucose-1-phosphate	Glycolysis	18.798	3.577±0.778	1.436±0.271	1.456	0.002
Lactic acid	Glycolysis	8.061	136.832±14.840	364.728±38.325	1.638	<0.001
Mannose	Fructose and mannose metabolism	20.119	654.393±71.645	246.199±43.934	1.463	<0.001
Pipecolinic acid	Lysine degradation	15.259	0.038±0.020	0.245±0.062	1.576	0.047
Ribitol	Pentose and glucuronate interconversions	16.676	2.449±0.341	0.890±0.104	1.849	<0.001
L-valine	Valine, leucine and isoleucine degradation	9.819	30.076±2.667	20.071±1.599	1.339	0.002

^a^Pathway was consulted from KEGG

^b^ R.T.: retention time

^c^The number of column Normal and RA mean the relative concentration to the internal reference and expressed with mean±SE

Several other different metabolites were also found in this study, including 5-methoxytryptamine, carnitine, pipecolinic acid, ribitol, beta-mannosylglycerate and diglycerol. 5-Methoxytryptamine is of tryptophan metabolism. Carnitine and pipecolinic acid are of lysine degradation, carnitine also was an essential factor in fatty acid metabolism in mammals and its main metabolic function was to transport fat into the mitochondria for oxidation. Ribitol was an end product of the reduction of ribose in human fibroblasts and can be cleared from the body without metabolic conversion. All these different metabolites have been reported in RA and other inflammatory diseases [22]. The metabolic pathway of beta-mannosylglycerate and diglycerol were unknown.

### Verification of different energy metabolism enzymes in RA patients and normal subjects by quantitative real-time PCR and western blot

**Fig 2:**
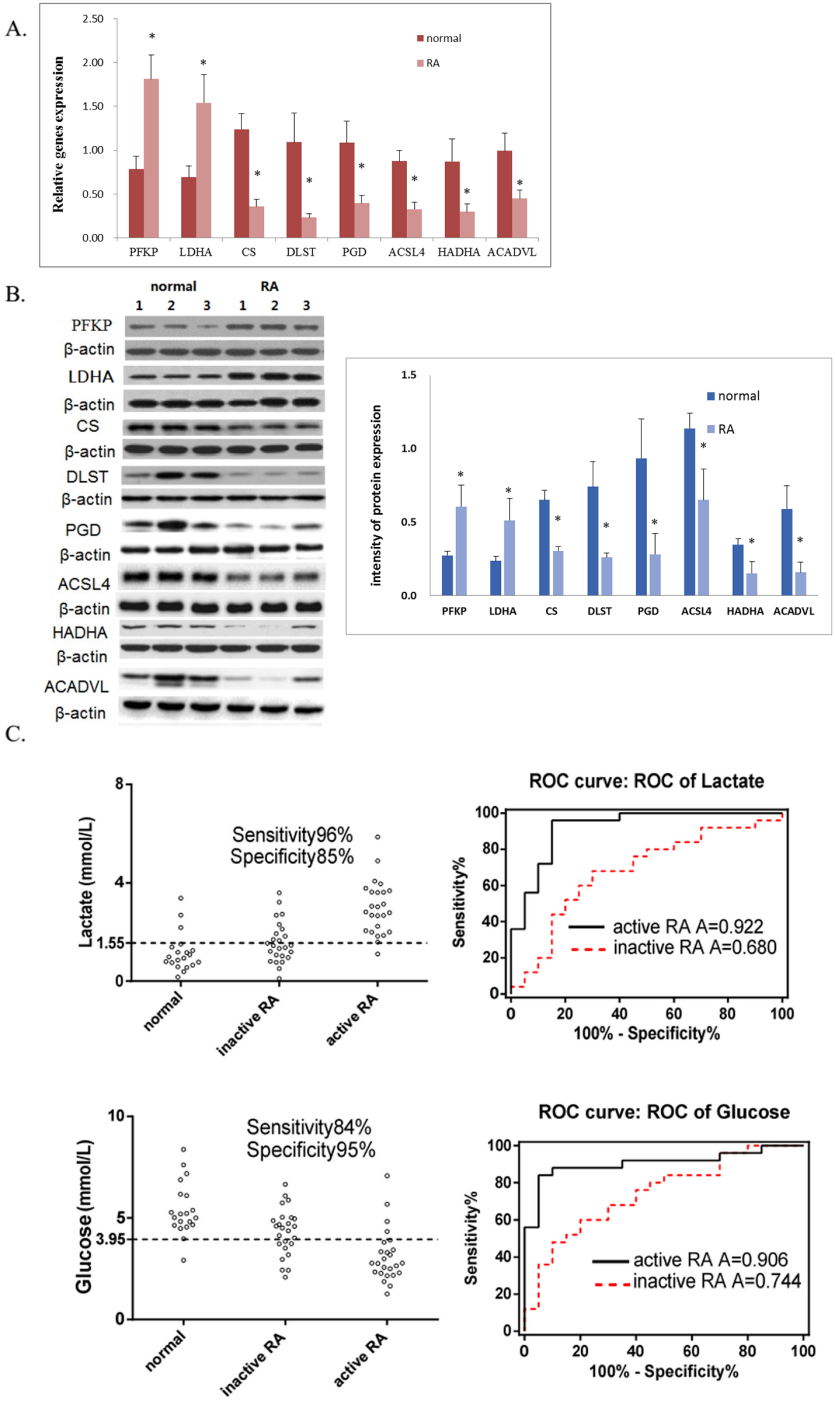
Verification of different metabolites and enzymes in synovial fluid and synovial tissues from RA patients and normal subjects. (A) Real-time PCR amplification was performed, the results were normalized for the amount of *β-actin* as internal control. Each value represents the average from 50 RA patients or 20 normal subjects. (B) Western blot of different enzymes. Protein samples were analyzed on 10% SDS–PAGE, followed by immunoblotting, left panel is the band image of enzymes from 3 RA patients and 3 normal subjects. The mean intensity of corresponding protein expression in RA patients (20 cases) and normal subjects (20 cases) was shown in the right panel. (C) The concentration of glucose and lactic acid were verified in synovial fluid from inactive RA patients (25 cases), active RA patients (25 cases) and normal subjects (20 cases) by colorimetric assay kit and ROC curve analysis. ROC curve analysis showed that the concentration of glucose and lactic acid in synovial fluid could be used as dependable biomarkers for the diagnosis of active RA, provided an AUC of 0.906 and 0.922. Sensitivity and specificity, which were determined by cut-off points, reached 84% and 96% in sensitivity and 95% and 85% in specificity, respectively.

46 enzymes of energy metabolism were identified in FLS cells from synovial membrane in our previous competitive proteomics study [17]. Among them 12 enzymes were different between RA patients and normal subjects. PFKP and P4HA1 were increased and the others were decreased in RA patients, including DLST, PGD, CS, ACO1, GFPT2, G6PD, MDH1, ACSL4, HADHA and ACADVL (Table 2). 7 of the 12 differentially expressed proteins were verified by real-time PCR and western blot in synovial tissues of RA patients and normal subjects. The real-time PCR result showed that the expression of PFKP and LDHA were elevated, and CS, DLST, PGD, ACSL4, HADHA and ACADVL were reduced in the synovial tissues of RA patients as compared to normal subjects (Fig 2A). The RA/normal ratio was 2.32, 2.23, 0.29, 0.22, 0.37, 0.38, 0.34 and 0.46, respectively. These results were strongly consistent with the results obtained by western blot, in which the RA/normal ratio was 2.21, 2.16, 0.47, 0.35, 0.30, 0.57, 0.44 and 0.27 (Fig 2B).

**Table 2:** Different enzymes in RA patients and normal subjects related to energy metabolism.

Gene name	ACC^a^	Protein name	EC^b^	Pathway name consulted from KEGG	Normal (mean±SE)	RA (mean±SE)	P
DLST	P36957	2-oxoglutarate dehydrogenase complex component E2, mitochondrial	2.3.1.61	Citrate cycle	0.483±0.059	0.227±0.053	0.032
PFKP	Q01813	6-phosphofructokinase type C	2.7.1.11	Glycolysis	0.506±0.018	0.891±0.053	0.012
PGD	P52209	6-phosphogluconate dehydrogenase, decarboxylating	1.1.1.44	Pentose phosphate pathway	1.084±0.101	0.341±0.032	0.002
CS	O75390	Citrate synthase, mitochondrial	2.3.3.1	Citrate cycle	0.643±0.042	0.290±0.080	0.017
MDH1	P40925	Malate dehydrogenase, cytoplasmic	1.1.1.37	Citrate cycle	1.064±0.057	0.695±0.033	0.005
ACO1	P21399	Cytoplasmic aconitate hydratase	4.2.1.3	Citrate cycle	0.294±0.034	0.044±0.002	0.018
GFPT2	O94808	Glucosamine—fructose-6-phosphate aminotransferase 2	2.6.1.16	Alanine, aspartate and glutamate metabolism	0.374±0.056	0.132±0.037	0.023
G6PD	P11413	Glucose-6-phosphate 1-dehydrogenase	1.1.1.49	Pentose phosphate pathway	0.643±0.061	0.435±0.028	0.036
ACSL4	O60488	Long-chain-fatty-acid—CoA ligase 4	6.2.1.3	Fatty acid degradation	0.602±0.081	0.056±0.003	0.021
P4HA1	P13674	Prolyl 4-hydroxylase subunit alpha-1	1.14.11.2	Arginine and proline metabolism	0.454±0.007	0.553±0.010	0.001
HADHA	P40939	Trifunctional enzyme subunit alpha, mitochondrial	4.2.1.17	Fatty acid degradation/Valine, leucine and isoleucine degradation	0.878±0.148	0.403±0.033	0.035
ACADVL	P49748	Very long-chain specific acyl-CoA dehydrogenase, mitochondrial	1.3.8.9	Fatty acid degradation	0.723±0.061	0.429±0.031	0.012

^a^ACC: Accession number in Uniprot

^b^EC: Enzyme commission number.

### Verification of different metabolites by colorimetric assay kit

To verify lactic acid and glucose as a desired biomarker for RA diagnosis, their concentrations were detected in synovial fluid from 25 inactive RA patients, 25 active RA patients and 20 normal subjects by colorimetric assay kit. The results were the same as previously identified results by GC-MS. ROC curve analysis showed that the concentration of glucose and lactic acid in synovial fluid could be used as dependable biomarkers for the diagnosis of active RA, provided an AUC of 0.906 and 0.922. Sensitivity and specificity, which were determined by cut-off points, reached 84% and 96% in sensitivity and 95% and 85% in specificity, respectively. The results show the better diagnosibility of glucose and lactic acid in synovial fluids of active RA (Fig 2C).

### The verification of HIF-1α regulates the expression of energy metabolism enzymes by HIF-1α knockdown

**Fig 3:**
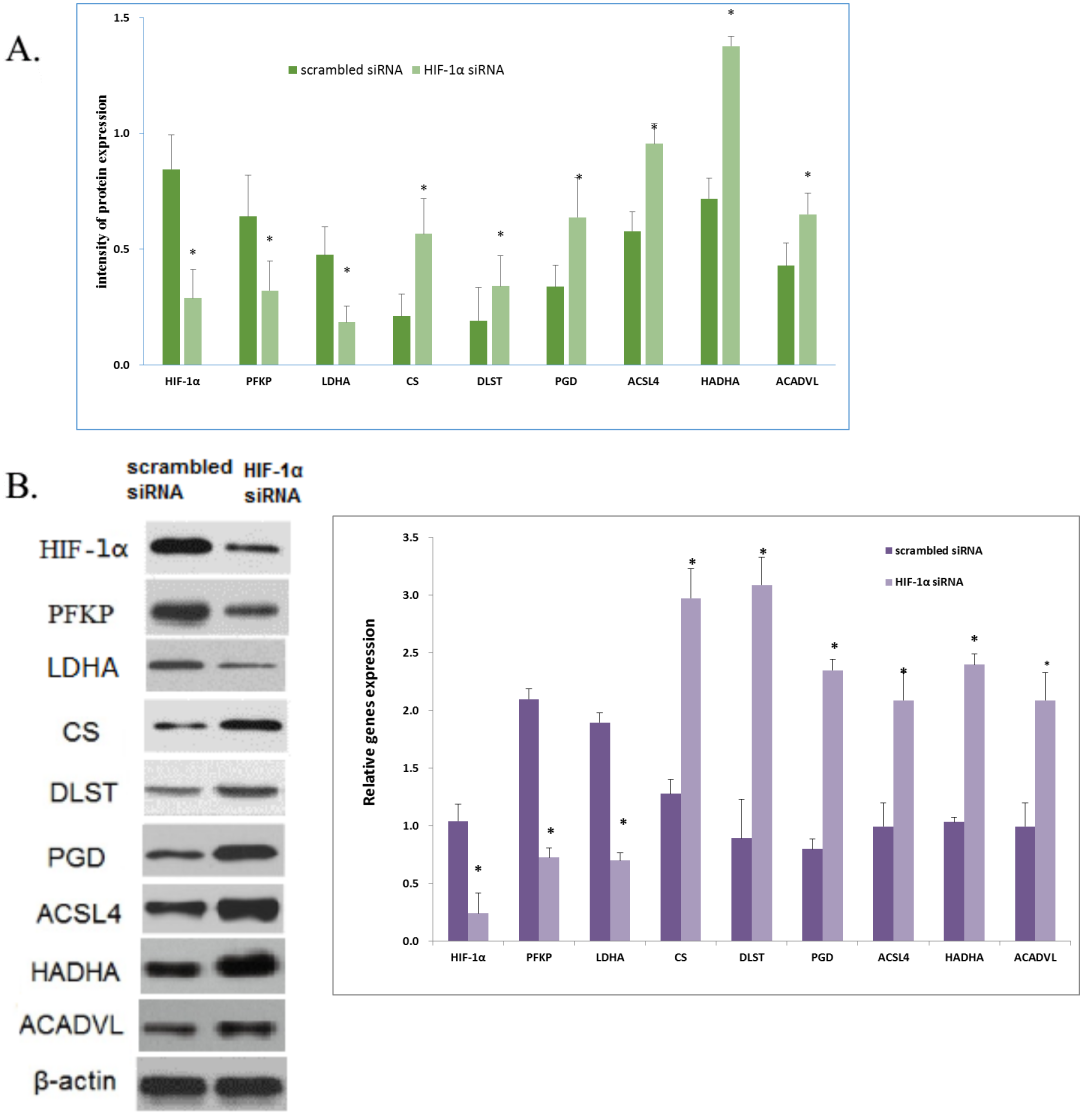
The effect of HIF-1α knockdown on the expression of PFKP, LDHA, CS, DLST, PGD, ACSL4, HADHA and ACADVL in FLS cells. (A) Real-time PCR of enzymes in FLS cells transfected with scrambled siRNA or HIF-1α siRNA for 24hr, β-actin was used as a loading control. (B) Western blot of enzymes in FLS cells transfected with scrambled siRNA or HIF-1α siRNA for 48hr, β-actin was used as a loading control. All experiments were performed at least in triplicates, the data are presented as mean ± SD, *p<0.05, compared with control, the asterisks represent significant differences.

To evaluate the effect of hypoxia on the expression of energy metabolism enzymes, HIF-1α knock-down experiments were performed by transfecting HIF-1α specific siRNA or scrambled siRNA into FLS cells by Lipofectamine 2000 (Invitrogen, USA). The real-time PCR result showed that the expression of HIF-1α PFKP and LDHA were reduced, and the expression of CS, DLST, PGD, ACSL4, HADHA and ACADVL were elevated in HIF-1α siRNA as compared to scrambled siRNA transfected FLS cells (Fig 2A). The HIF-1α siRNA/scrambled siRNA ratio was 0.23, 0.35, 0.37, 2.32, 3.46, 2.95, 2.10, 2.32 and 2.10, respectively. These results were strongly consistent with the results obtained by western blot, in which the HIF-1α siRNA/scrambled siRNA ratio was 0.34, 0.50, 0.39, 2.69, 1.78, 1.88, 1.65, 1.92 and 1.51, respectively (Fig 3A).

## Discussion and Conclusion

**Fig 4:**
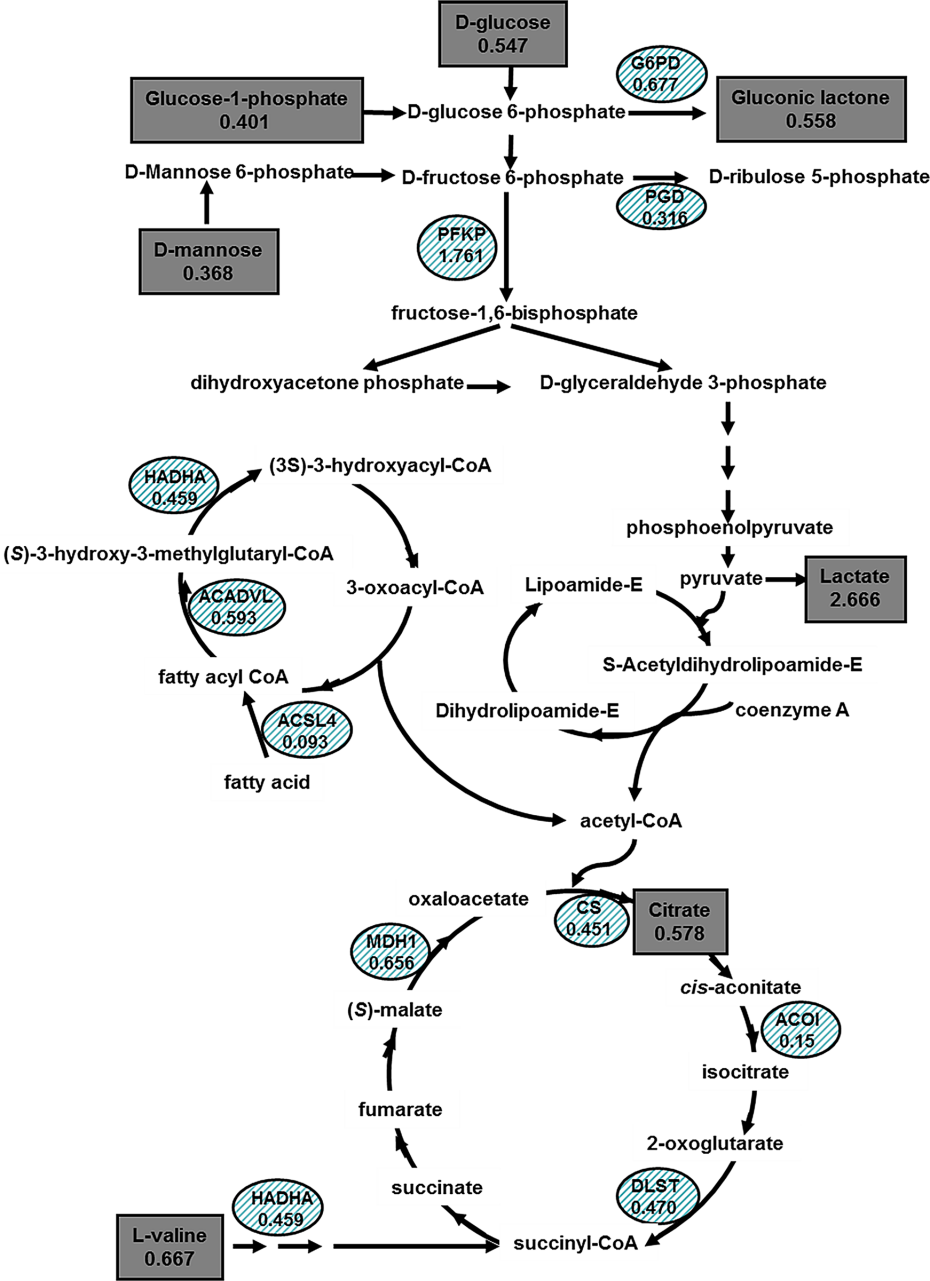
Energy metabolism profiles obtained from RA patients and normal subjects based on comparative proteomics and metabolomics study. The different metabolites and enzymes are marked with a rectangle and a circle, respectively. The number means the ratio of RA to normal. Arrow means the direction of reaction.

Rheumatoid arthritis is an autoimmune disease involving multiple molecules and pathways. Given the integrated nature of systemic metabolism may provide a better understanding of the disease-associated changes [23,24]. Glucose metabolism not only provides energy for physical activity but also mediates a variety of physiological processes through the formation of complex signaling networks. Cells need energy supplied by ATP, which is mainly produced by the anaerobic decomposition and aerobic oxidation pathways [16,25]. Maximum aerobic oxidation of glucose per molecule can generate 32 ATP molecules. Anaerobic decomposition can produce two ATP molecules [11], which is significantly lower than the number of ATP molecules produced by aerobic oxidation [12]. Then, what is the main energy metabolism in RA joints? An overview of the present metabolomic study and our previous proteomic result indicates the possibility of clarifying the exact mechanism underlying the observed perturbation between RA patients and healthy controls, and revealing the energy metabolism of RA (Fig 4).

### Anaerobic catabolism is enhanced in RA patients

Synovial fluid is the inherent ingredient of articular cavity from plasma and plays a role in lubricating the joints and nourishing the articular cartilages. Our identification and verification results show that the glucose concentration was decreased and the lactic acid concentration was increased in synovial fluid of RA patients, which is consistent with the findings of Naughton et al [18,26] and Young et al [15], showing that the concentration of lactic acid was elevated in inflammatory synovial fluid. High lactic acid level has also been reported in synovial fluid of seropositive RA patients as compared to seronegative RA patients [25]. Although the cause remains unclear but it is known that lactic acid is the end product of anaerobic catabolism, and PFKP, an important key enzyme of glycolysis, was significantly over-expressed in RA patients in our previous proteomic study and this verification experiment. Henderson et al [27] also found that the activity of glyceraldehyde 3-phosphate dehydrogenase and lactic acid dehydrogenase, the major enzymes of the glycolytic pathway, also increased in RA synovial cells, indicating that glycolytic activity was increased in synovial tissues of RA patients. In the present study we also found that the expression of lactic acid dehydrogenase was higher in RA synovial tissue and decreased after HIF-1α knockdown. Because increased glycolytic activity in RA can lead to lactic acidosis, the acidic microenvironment might disrupt abnormal cell differentiation in synovial tissues of individuals with RA [28]. At the same time, the levels of glucose-1-phosphate and D-mannose as donors of glycolysis were decreased. The increased demand of energy in inflammation may increase the consumption of glucose in RA patients as compared to the normal controls, which may be the cause of decrease in glucose, Glucose-1-phosphate and D-mannose levels. The increased consumption of glucose increases the production of lactic acid. It is common knowledge that the aerobic oxidation pathway produces more energy than anaerobic catabolism under the same condition of glucose consumption, how about aerobic oxidation in RA patients?

### Aerobic oxidation is weakened in RA patients

The citric acid cycle is the central metabolic pathway for all aerobic processes. The cycle provides the complete oxidation of acetyl-CoA derived from fats, carbohydrates and amino acids into carbon dioxide and capturing the released energy as reductive power in the form of NADH and FADH_2_. Our preliminary proteomic result showed that enzymes of the TCA cycle (including CS, MDH1, ACO1 and DLST) were down-regulated in the synovial fluid of RA patients. Among them, CS and DLST are the rate-limit enzymes of the TCA cycle. The identification results show that the expression of CS and DLST was significantly decreased in RA patients. In consistence with CS decrease in the RA synovial fluid, in our study the level of citric acid was significantly decreased in the synovial fluid of RA patients [29]. The down-regulation of CS and DLST decreases the ATP production from the aerobic oxidation process.

### Other factors that influence sugar catabolism

Our preliminary proteomic result also showed that fatty acid beta oxidation enzymes, including ACSL4, HADHA and ACADVL, were decreased in the RA synovial membrane. ACSL4 catalyzes the formation of FA-CoA, which is the pre-step reaction for β-oxidation of fatty acids and plays a crucial role in fatty acid degradation. ACADVL and HADHA catalyze the yield of acetyl CoA in the final step of mitochondrial beta-oxidation of long chain fatty acids. On the other hand, the concentration of L-valine was decreased, which is known to be the substrate donor of the TCA cycle.

Why does the inflamed rheumatoid joint favor the anaerobic catabolism to be the energy source? Hitchon et al [30] recently confirmed that anaerobic metabolism is evident with progressively increasing degrees of RA synovial inflammation and vascularity. Hollander et al [31] observed the up-regulation of HIF-1α in synovial CD68^+^ macrophages prepared from biopsy samples from patients with RA. Giatromanolaki et al [32] also observed increased expression of HIF-1α and HIF-2α in the synovial lining and stromal cells in subjects with the disease. Activation of the HIF signal cascade leads to extensive changes in gene expression, which allows cells, tissues and organisms to adapt to reduced oxygenation. These changes include enhanced glucose uptake by increasing the expression of glucose transporters GLUT1 and GLUT3, increased the expression of glycolytic enzymes (regulating the levels of hexokinase II, 3-GAPDH, lactate dehydrogenase and mitochondrial cytochrome oxidase in the inflammatory synovium to enhance glycolytic activity) and angiogenic factors [33–37]. In this study, the expression of PFKP (key enzymes of glycolysis [38]) and LDHA (catalyzes the conversion of pyruvate to lactic acid, which is the final product of anaerobic catabolism) were decreased and the expression of CS, DLST, PGD, ACSL4, HADHA and ACADVL were increased after HIF-1α knockdown, which revealed that HIF was a key factor in the regulation of anaerobic catabolism. The increased glycolytic activity regulated by HIF-1α in RA synovial tissues can elevate the production of lactic acid and pyruvate, which drives abnormal angiogenesis and pannus formation in the diseased joints. Lactic acid was also detected in the synovial fluid of patients with a variety of non-septic arthritis, including RA, reactive arthritis and gout [39]. These observations are consistent with the markedly hypoxic status of the inflamed rheumatoid joint. In the inflamed RA joint, the synovial tissue is highly infiltrated by CD4^+^ T cells, B cells, and macrophages, and the intimal lining becomes hyperplastic owing to the increased number of macrophage-like and FLSs [40]. Proliferation of synoviocytes and accumulation of infiltrates contribute to an increase in the consumption of oxygen. In addition, the hyperplastic intimal synovial lining causes hypoperfusion because the oxygen diffusion limit is exceeded (100–200 mm) [41].

In summary, enhanced anaerobic catabolism and weakened aerobic oxidation promote energy generation from oxidative phosphorylation to anaerobic glycolytic metabolism. Hypoxia-HIF induced energy metabolism disorder plays an important role in the pathogenesis of RA, low glucose and high lactic acid concentration in synovial fluid maybe the potential biomarker of RA.

## Supporting Information

S1 Table A: Demographic characteristic of the RA patients and healthy controls of identification experiment; B: Demographic characteristic of RA patients and healthy controls for verification experiment. (DOC)

S2 Table Primer sequences for Real-Time PCR Analysis. (DOC)

S3 Table Identified metabolites in synovial fluid of RA patients and normal subjects. (DOC)
