# Supplementary material for: Energy Metabolism Disorder as a Contributing Factor of Rheumatoid Arthritis: A Comparative Proteomic and Metabolomic Study
Source: PLoS One. 2015 Jul 6;10(7):e0132695. doi: 10.1371/journal.pone.0132695 (PMC4492520; doi:10.1371/journal.pone.0132695)
Supplement: S1 Table — (DOC) [file pone.0132695.s001.doc]

Table S1A Demographic characteristic of the RA patients and healthy controls of identification experiment.

|  | **Normal** | **RA** |
| --- | --- | --- |
| **Number** | 10 | 25 |
| **Age(years)** | 45.2±8.01 | 49.64±8.43 |
| **Male/Female** | 4/6 | 11/14 |
| **Serum CRP(mg/dl)** | 0.18±0.05 | 2.16±1.63 |
| **ESR(mm/h)** | 9.83±3.06 | 42.83±27.53 |
| **Duration of disease** | NA | 5.96±5.31 |
| **NSAIDs usage (%)** | NA | 79.2 |
| **DMARDs usage (%)** | NA | 64.1 |

Values are expressed as Mean ± S.D.

NA: not applicable.

Table S1B Demographic characteristic of RA patients and healthy controls for verification experiment.

|  | **Normal** | **Inactive RA ( DAS28<3.2)** | **Active RA**  **( DAS28>3.2 )** |
| --- | --- | --- | --- |
| **Number** | 20 | 25 | 25 |
| **Age(years)** | 47.2±9.91 | 53.64±6.32 | 57.56±9.75 |
| **Male/Female** | 8/12 | 11/14 | 10/15 |
| **Serum CRP(mg/dl)** | 0.19±0.09 | 0.20±0.07 | 1.37±0.89 |
| **DAS28 score** | 0.94±0.41 | 2.57±1.35 | 5.38±3.93 |
| **ESR(mm/h)** | 9.80±2.67 | 28.35±13.41 | 57.36±29.41 |
| **Duration of disease(years)** | NA | 4.1±2.7 | 8.3±4.3 |
| **NSAIDs usage (%)** | NA | 78.3 | 81.3 |
| **DMARDs usage (%)** | NA | 63.5 | 65.1 |

Values are expressed as mean ± S.D.

NA: not applicable.
