# Supplementary material for: Energy Metabolism Disorder as a Contributing Factor of Rheumatoid Arthritis: A Comparative Proteomic and Metabolomic Study
Source: PLoS One. 2015 Jul 6;10(7):e0132695. doi: 10.1371/journal.pone.0132695 (PMC4492520; doi:10.1371/journal.pone.0132695)
Supplement: S2 Table — (DOC) [file pone.0132695.s002.doc]

Table S2 Primer sequences for Real-Time PCR Analysis

|  | **Genbank** | |
| --- | --- | --- |
| gene | Forward | Reverse |
| *hif-1**α* | 5΄-gattcaagtggtcttcctgcttcagc-3΄ | 5΄-gggactcatcccaggcggg-3΄ |
| *pfkp* | 5’- agatccataaggaggccgtg-3’ | 5’-agaacgaaggtcctctggtg-3’ |
| *ldha* | 5'-tgcctgtatggagtggaa-3' | 5'-cctgcttgtgaacctctt-3' |
| *cs* | 5’-ttgtcttgttcttgcagccc-3’ | 5’-ccatgttgctgcctgaaagt-3’ |
| *dlst* | 5’-tggtgaaacggatgaggtca -3’ | 5’- atgaatcccagtggctccaa-3’ |
| *acsl4* | 5’-agcactgaacctgggaaaga-3’ | 5’-tcagcaacagcaaacagacc-3’ |
| *pgd* | 5’-ttatttgtggggagcggagt-3’ | 5’-tctttgttccctcctggcat-3’ |
| *hadha* | 5’-tgacccgaagaagctgaatt-3’ | 5’-tagctacatccacaccaact-3’ |
| *acadvl* | 5’-cgctttctgctcaaccgagc-3’ | 5’-tgaagatgtctgctaggccc-3’ |
| *β-actin* | 5'-atggtggtatgggtcagaag-3' | 5'-tgggctggggtgttgaaggtc-3' |
