# Supplementary material for: Energy Metabolism Disorder as a Contributing Factor of Rheumatoid Arthritis: A Comparative Proteomic and Metabolomic Study
Source: PLoS One. 2015 Jul 6;10(7):e0132695. doi: 10.1371/journal.pone.0132695 (PMC4492520; doi:10.1371/journal.pone.0132695)
Supplement: S3 Table — (DOC) [file pone.0132695.s003.doc]

Table S3 Identified metabolites in synovial fluid of RA patients and normal subjects

| Number | Metabolite | RT^a^ | Mean of N^b^ | Mean of RA | VIP | P value | RA/N^b^ |
| --- | --- | --- | --- | --- | --- | --- | --- |
|  | 1,5-Anhydroglucitol | 19.099 | 6.450 | 5.656 | 0.044 | 0.581 | 0.877 |
|  | 2-Amino-1-Phenylethanol | 12.423 | 1.368 | 1.020 | 0.852 | 0.235 | 0.745 |
|  | 2-Deoxyerythritol | 13.365 | 0.473 | 0.366 | 0.173 | 0.412 | 0.775 |
|  | 2-Hydroxybutanoic acid | 8.759 | 4.610 | 3.307 | 0.411 | 0.146 | 0.717 |
|  | 2-Hydroxypyridine | 8.030 | 0.596 | 0.286 | 0.783 | 0.338 | 0.481 |
|  | 3-Hydroxybutyric acid | 9.133 | 12.364 | 10.116 | 0.549 | 0.549 | 0.818 |
|  | 5-Methoxytryptamine | 19.264 | 1.006 | 0.727 | 1.115 | 0.022 | 0.723 |
|  | Alanine | 8.530 | 40.315 | 30.787 | 0.905 | 0.188 | 0.764 |
|  | Aminomalonic acid | 13.118 | 2.036 | 1.262 | 0.556 | 0.158 | 0.620 |
|  | Aspartic acid | 13.778 | 1.117 | 1.574 | 1.080 | 0.186 | 1.409 |
|  | beta-Mannosylglycerate | 29.545 | 0.557 | 1.272 | 1.269 | 0.035 | 2.286 |
|  | Carnitine | 16.956 | 0.011 | 0.091 | 2.020 | <0.001 | 8.254 |
|  | Cis-gondoic acid | 24.207 | 0.183 | 0.217 | 1.361 | 0.694 | 1.184 |
|  | Citric acid | 18.471 | 21.117 | 12.218 | 1.499 | 0.001 | 0.579 |
|  | Conduritol b epoxide | 22.762 | 12.653 | 8.846 | 0.989 | 0.038 | 0.699 |
|  | Creatine | 14.518 | 1.353 | 1.429 | 0.072 | 0.747 | 1.056 |
|  | Creatine degr | 9.979 | 1.021 | 0.727 | 0.504 | 0.122 | 0.712 |
|  | Cycloleucine | 11.339 | 0.212 | 0.150 | 0.793 | 0.412 | 0.709 |
|  | Diglycerol | 16.770 | <0.001 | 0.189 | 1.512 | 0.032 | 5580.456 |
|  | Ethanolamine | 15.012 | 0.320 | 0.228 | 0.918 | 0.137 | 0.714 |
|  | Fructose | 19.291 | 0.233 | 0.150 | 0.611 | 0.064 | 0.645 |
|  | Fucose | 11.730 | 0.036 | 0.038 | 0.774 | 0.939 | 1.047 |
|  | Gluconic lactone | 20.792 | 7.277 | 4.065 | 1.189 | 0.012 | 0.559 |
|  | Glucose | 19.545 | 21.640 | 11.846 | 1.187 | 0.006 | 0.547 |
|  | Glucose-1-phosphate | 18.798 | 3.577 | 1.436 | 1.456 | 0.002 | 0.402 |
|  | Glutamine | 17.839 | 0.940 | 0.520 | 0.734 | 0.076 | 0.553 |
|  | D-Glyceric acid | 11.135 | 2.079 | 4.059 | 0.447 | 0.596 | 1.953 |
|  | Glycerol | 10.394 | 39.082 | 28.287 | 0.860 | 0.186 | 0.724 |
|  | Glycine | 11.003 | 21.733 | 13.825 | 0.964 | 0.035 | 0.636 |
|  | Lactic acid | 8.061 | 136.832 | 364.728 | 1.638 | <0.001 | 2.666 |
|  | Leucine | 10.478 | 15.551 | 10.502 | 0.480 | 0.001 | 0.675 |
|  | Lysine | 20.304 | 3.410 | 2.580 | 0.985 | 0.017 | 0.757 |
|  | Maleimide | 8.633 | 0.000 | 0.199 | 0.974 | 0.064 | 5888.096 |
|  | Malonic acid | 21.496 | 0.897 | 0.600 | 0.603 | 0.023 | 0.669 |
|  | Mannose | 20.119 | 654.393 | 246.199 | 1.463 | <0.001 | 0.376 |
|  | Methionine | 13.932 | 0.585 | 0.472 | 0.908 | 0.060 | 0.808 |
|  | N-Acetyltryptophan | 24.191 | 0.495 | 0.468 | 0.269 | 0.609 | 0.945 |
|  | N-Methyl-DL-alanine | 9.303 | 1.984 | 1.116 | 0.702 | 0.021 | 0.562 |
|  | Norleucine | 10.765 | 5.824 | 3.976 | 0.507 | 0.003 | 0.683 |
|  | Ornithine | 18.551 | 1.523 | 1.808 | 0.485 | 0.308 | 1.187 |
|  | Oxalic acid | 22.845 | 27.201 | 18.971 | 0.574 | 0.124 | 0.697 |
|  | Oxoproline | 14.029 | 13.190 | 11.799 | 0.362 | 0.420 | 0.894 |
|  | Pentadecanoic acid | 22.322 | 1.529 | 1.254 | 0.613 | 0.352 | 0.820 |
|  | Phenylalanine | 14.617 | 1.927 | 1.533 | 0.791 | 0.162 | 0.795 |
|  | Phosphate | 10.458 | 3.371 | 2.284 | 1.403 | 0.310 | 0.677 |
|  | Pipecolinic acid | 15.259 | 0.038 | 0.245 | 1.576 | 0.047 | 6.373 |
|  | Proline | 10.934 | 14.948 | 13.715 | 0.439 | 0.531 | 0.918 |
|  | Ribitol | 16.676 | 2.449 | 0.891 | 1.849 | <0.001 | 0.364 |
|  | Serine | 11.543 | 2.389 | 2.119 | 0.295 | 0.410 | 0.887 |
|  | Threonine | 10.810 | 0.115 | 0.191 | 0.767 | 0.165 | 1.663 |
|  | Tyrosine | 20.637 | 1.270 | 1.009 | 0.964 | 0.021 | 0.795 |
|  | Urea | 10.135 | 88.716 | 48.636 | 0.577 | 0.003 | 0.548 |
|  | Valine | 9.819 | 30.076 | 20.071 | 1.339 | 0.002 | 0.667 |
|  | Zymosterol | 27.467 | 2.593 | 2.871 | 0.070 | 0.823 | 1.107 |
|  | Glycocyamine | 11.532 | 0.001 | 0.013 | 1.003 | 0.068 | 18.015 |
|  | L-Threose | 16.063 | 2.982 | 3.719 | 0.161 | 0.434 | 1.247 |
|  | L-cysteine | 14.360 | 1.952 | 1.285 | 0.444 | 0.166 | 0.658 |
|  | L-Allothreonine | 11.889 | 23.473 | 20.086 | 0.704 | 0.195 | 0.856 |

^a^RT: retention time

^b^N: normal subjects
